# Effects of humate, *Yucca*, and humate-*Yucca* combination supplementation on growth performance, gut health, and immune response in broiler chickens

**DOI:** 10.3389/fvets.2025.1692212

**Published:** 2025-10-17

**Authors:** Selim Mert

**Affiliations:** Department of Animal Science, Faculty of Agricultural, Ege University, Izmir, Türkiye

**Keywords:** broiler, growth performance, gut microbiota, natural feed additives, antibiotic alternatives

## Abstract

This study evaluated the effects of dietary humate (300 mg/kg), *Yucca* (100 mg/kg), and their combination (300 mg/kg H + 100 mg/kg Y) on growth performance, intestinal microbiota, ileal digestibility, and blood parameters in broiler chickens. A total of 240 one-day-old Ross 308 broiler chicks were randomly divided into four groups and reared for 42 days. Growth performance significantly improved in the supplemented groups. Body weight gain and feed conversion ratio were better in the humate and *Yucca* groups, with the highest improvement observed in the combination group (HY), which showed a feed conversion ratio close to 1.50 compared to 1.66 in the control. In gut microbiota, *E. coli* counts decreased notably from over 5.3 log CFU/g in the control to around 2.0 in the HY group. Lactic acid bacteria and total bacterial counts increased by approximately 1.2–2.3 log CFU/g with supplementation. Ileal digestibility also improved: crude fat digestibility increased from 85.8% in the control to 88.1% in HY; crude protein from 85.1 to 87.1%; and organic matter from 83.4 to 85.5%. Blood analysis showed higher immune markers in supplemented groups. IgA levels increased from 9.5 mg/dL to nearly 11.0 mg/dL, and IgM from 7.1 to 8.3 mg/dL. Thyroid hormones T3 and T4 also increased slightly, indicating improved metabolic activity. In conclusion, humate and *Yucca* especially in combination positively affected gut health, nutrient utilization, immune function, and overall performance in broiler chickens, suggesting their potential as effective natural feed additives.

## 1 Introduction

Since the 1950s, various additives have been added to feed in order to support the immune system, increase nutrient digestibility, achieve maximum yield performance, and improve product quality in broiler genotypes used in conventional chicken meat production. In this context, natural products have come to the fore among feed additives in recent years due to increasing consumer sensitivity regarding food safety. *Yucca* (*Yucca schidigera*) extract and humate are natural multifunctional products that positively affect animal health, productivity performance, and the quality and safety of the products obtained. Considering that many endemic animal diseases such as avian influenza have turned into pandemics worldwide and that the use of antibiotics has been banned in European Union countries and Turkey (1), the use of multifunctional feed additives such as *Yucca* and humate has become increasingly significant. To date, studies have been conducted on the usability of these products in broiler chicken nutrition, and their effects and mechanisms have been examined separately. Due to the close relationship between performance, intestinal health, and the immune system in broiler chicken production, it is currently recommended to add feed additives such as enzymes, probiotics, prebiotics, synbiotics, organic acids, and plant-based products to feed or water. In this context, numerous studies have been conducted in recent years to determine the antioxidant, antimicrobial, immune-supporting, and growth-promoting effects of phytobiotics. *Yucca shidigera.*

*Yucca shidigera* is a desert plant that contains two important compounds. The first of these is phenols, which have an ammonia-binding effect. The second is steroidal saponins, which, with their surface-active properties, help the plant minimize the negative effects of stress by retaining water and nutrients within the plant (2). Depending on its saponin content, *Yucca* exhibits ammonia-binding, urease activity-inhibiting, intestinal epithelial cell surface tension-reducing, antiprotozoal, antibacterial, antifungal, and antioxidant properties (3). Due to their water- and oil-soluble properties, saponins play a significant role in the formation of micelles during fat absorption and also facilitate the passage of other nutrients through the intestinal epithelium (4). Additionally, this substance binds to the cell membranes of pathogenic microorganisms in the intestines, causing their death and exhibiting antibacterial effects (5). Saponins increase nutrient absorption by reducing surface tension in the intestines and triggering the development of intestinal villi, thereby having a positive effect on performance (6). These properties of the *Yucca* plant have led to numerous promising studies on its use in the nutrition of broiler chickens.

In a study conducted by Ayoub et al. (7), *Yucca* extract was added to drinking water, and broiler chickens were provided with this water for 8 h a day. At the end of the study, the researchers reported that the group using *Yucca* extract had a significant decrease in ammonia concentration in the litter, a significant decrease in *total bacteria* and *E. coli* counts in the manure, an increase in antioxidant enzyme levels, an increase in immunoglobulin M and G levels, and positive results in fat oxidation parameters. In addition to all of these positive results, the researchers determined that feed utilization efficiency had improved significantly and stated that *Yucca* extract could be successfully used in broiler chicken production. Sun et al. (8) added 100, 200, and 300 mg/kg of *Yucca* extract to broiler chicken feed between days 14 and 42. The researchers found that there were no significant differences between the groups in terms of live weight gain, feed consumption, and feed utilization at day 28, but that catalase (CAT) enzyme activity and gene expression had increased significantly. Between days 28 and 42 of the experiment, daily live weight gain and feed efficiency improved in the group receiving 100 mg/kg of *Yucca* extract. Additionally, the addition of 200 and 300 mg/kg of *Yucca* extract was found to positively affect the antioxidant defense system and lipid peroxidation.

In a study conducted by Bafundo et al. (9), the effects of the coccidiosis vaccine were examined in broiler chickens fed a combination of quillaja and *Yucca*. It was determined that *Yucca* extract containing saponin functions synergistically with the coccidiosis vaccine to strengthen immunity. Begum et al. (10), who used caprylic acid and *Yucca* extract together, reported that live weight gain and feed conversion ratio improved significantly, relative organ weights and lymphocyte levels increased, and the number of *E. coli* in manure decreased. In a study combining curcumin, one of the main active components of turmeric, with *Yucca* extract, comparisons were made between antibiotic and coccidiostatic additives (11). In the study, 100 mg/kg of curcumin, 250 mg/kg of *Yucca* extract, and a combination of the two at the same doses were added to mixed feed. The results showed improvements in parameters such as blood values and lipid oxidation in the group treated with curcumin. Although protein peroxidation decreased in the group using only *Yucca* extract, the researchers recommended the combined use of these two substances to enhance growth.

Humates are formed by the decomposition of plant residues in the soil and contain organic acids such as humic, fulvic, and ulmic acids in their structure (12). These organic acids optimize the pH value of the digestive tract and thus prevent the proliferation of pathogenic microorganisms. Additionally, they increase the absorption of calcium and various trace elements (13, 14). The humic and fulvic acids present in humate chelate toxic elements such as mercury and lead, rendering them harmless, and also prevent viral particles from attaching to cell surfaces, thereby exhibiting antiviral effects (15). Humic substances form a protective membrane on the surface of the intestinal epithelium, preventing the formation of lesions caused by any agent and promoting improved epithelial cell development (16).

It has been reported that adding 0.1 and 0.3% humic acid to aflatoxin-contaminated feed improves hematological parameters in broiler chickens, and this effect is attributed to the binding effect of humic acid (17). Ozturk et al. (18) added 0, 150, 300, and 450 ppm of humic acid to broiler chicken mixed feed in their study, and at the end of 42 days, live weight gain increased in the 300 ppm group while it decreased in the 450 ppm group. Carcass yield reached the highest level in the group fed with feed containing 150 ppm humic acid. In another study, 5 g/kg of natural humic substances and 7 g/kg of sodium humate were added separately to broiler chick feed and chicken growth and finishing feed (19). At the end of the study, no significant difference was observed in slaughter weights, but the treatment groups utilized the feed more efficiently than the control group. This study concluded that the effects of different humate sources were similar. Korsakov et al. (20) added a commercially available water-soluble humate product at a level of 0.5 mL/L to the drinking water of broiler chickens in their study. The researchers observed that the addition of humate resulted in a 9.9% increase in carcass yield. Nagaraju et al. (21) added 0.5, 0.75, and 1 g/kg of humic acid to broiler chicken compound feed. According to the study data, feed utilization rate improved in the group receiving 0.75 g/kg humic acid at the end of the period. The proportional values of carcass parts and internal organ weights were not affected by the addition of humic acid fed broiler chickens with mixed feed containing 0.5, 1.0, and 2.5% humate. The researchers reported that humate had no toxic effects and improved feed utilization despite reducing live weight. Hassan et al. (22) conducted a study with four groups of broiler chickens, adding a commercial humate preparation at levels of 0.1, 0.25, and 0.40% to the basal mixed feed. Humate added at a level of 0.25% to the feed positively affected live weight, feed utilization, carcass characteristics, and intestinal microbiology in broiler chickens, increased intestinal pH, and reduced blood protein levels while having no effect on blood lipids and cholesterol levels.

This study was conducted to investigate the effects of the combined use of humate and *Yucca*. Although the literature review reveals numerous studies highlighting the significant effects of humate and *Yucca* individually, no studies have been found that explore their combined use. Therefore, this study aims to shed light on the changes that may occur as a result of the joint application of humate and *Yucca*.

## 2 Materials and methods

### 2.1 Animal ethics

The experimental protocol and all procedures were conducted in strict accordance with ethical standards and approved by the Local Ethics Committee for Farm Animal Experiments at Ege University Faculty of Agriculture (Approval No: 2023/002). The committee ensures that the use and care of research animals is ethical and humane.

### 2.2 Animals, housing, and experimental design

In this study, a total of 240 one-day-old Ross 308 broiler chicks with an initial live weight of 38.38 ± 0.44 g were randomly divided into 4 groups. Each group consisted of five replicate groups, each containing 12 chicks. The study groups were as follows: Control (C) basal diet, 300 mg/kg humate + basal diet (H), 100 mg/kg *Yucca* + basal diet, 300 mg/kg humate + 100 mg/kg *Yucca* + basal diet (HY). Chickens were provided with feed and water ad libitum until 42 days of age. The feed was prepared to be isocaloric and isonitrogenous. The initial and final diets (Table 1) were administered on days 1 and 21, and days 22 and 42, respectively. The compound feeds were prepared by mixing the powdered raw materials and feed additives using a mixer located in the mixing facility. The ambient temperature was initially set to 32 ± 1 °C and gradually reduced to 24 ± 1 °C. The lighting program consisted of 23 h of light and 1 h of darkness (for 42 days), and the relative humidity was maintained at 65%. Each 1.4 m × 1.2 m floor cage was equipped with wood shavings as bedding material, a round feeder, and four to five nipple drinkers to ensure adequate access to feed and water. The vaccination schedule of the company from which the chicks were sourced was followed. Newcastle vaccination was administered nine days after hatching, and Gumboro vaccination was applied on the 11th day. In the study, the usage doses of humate and *Yucca* were determined by taking averages based on the literature review. In addition, the amounts of the additives were recommended by the companies from which they were sourced. Each kg of humate contained 160 mg polmeric polyhydroxy acid (humic, fulvic, ulmic and humatomelanic asids). 663.3 SiO_2_ and other minerals (Mn 50 mg, Zn 60 mg, Fe 60 mg, Cu 5 mg, Co 0.2 mg, I 1 mg, Se 0.5 mg, and Al Na, K, Mg and P in trace amounts). *Yucca schidigera*, in 100% pure powder form, was sourced from Vizyonmix BF2338 Biopowder. Saponins are generally amorphous and colorless molecules, but they have types that are crystalline and white in color; they are soluble in polar solvents such as water, ethyl alcohol, and methyl alcohol (39). Structurally, saponins consist of two parts: the glycan and the aglycone, also known as sapogenin, and depending on the structure of the aglycone part, they are grouped into two categories: steroidal or triterpenoidal saponins.

**Table 1:** Ingredient composition and nutrient content of the diets.

	Starter 0–21 days (g/kg diet)	Finisher 22–42 days (g/kg diet)
Corn	440.04	490.00
Soybean meal	160.68	135.00
Full-fat soybean	154.00	150.00
Wheat	55.00	50.00
Sunflower meal	100.00	100.00
DCP	8.75	8.20
Marble	9.04	9.18
Methionine	3.49	1.56
Lysine	2.00	0.93
Salt	4.32	4.55
Vegetable oil	55.68	32.08
Vitamin and mineral premix	3.50	3.50
Calculated content		
Metabolic energy (Kcal/kg diet)	3,200	3,100
Dry matter (%)	89.61	89.32
Crude protein (%)	21.70	20.50
Crude fat (%)	10.05	7.82
Crude fiber (%)	5.34	5.35
Crude ash (%)	5.77	5.78
Ca (%)	0.70	0.70
P (%)	0.35	0.35
Sodium (%)	0.20	0.20

Vitamin–mineral premix 1 provided per kg of the complete diet: vitamin A, 5,000,000 IU; vitamin D3, 1,200,000 IU; vitamin E, 140,000 mg; vitamin K3, 1800 mg; vitamin B1, 1800 mg; vitamin B2, 3,000 mg; vitamin B6, 2080 mg; vitamin B12, 12 mg; D-biotin, 60 mg; folic acid, 1,000 mg; niacin, 24,000 mg; calcium-D pantothenate, 5,600 mg; manganese (manganese oxide), 32,000 mg; iron (iron sulfate monohydrate), 20,000 mg; copper (copper sulfate pentahydrate), 2000 mg; iodine (calcium iodate anhydride), 400 mg; cobalt (cobalt carbonate monohydrate), 60 mg; zinc (zinc oxide), 30,000 mg; selenium (sodium selenite), 40 mg.

### 2.3 Growth performance data collection

The body weight (BW) and feed intake (FI) of the male broilers were evaluated on a replicate cage basis on days 1, 21, and 42 of the study. These measurements were employed to calculate body weight gain (BWG) over three distinct periods: days 1 to 21, days 22 to 42, and days 1 to 42. Additionally, feed efficiency was assessed by determining the FCR based on the FI and BWG data.

### 2.4 Carcass characteristics and internal organs

On day 42, 12 broilers were selected from each treatment group, closely matching the average body weight (two birds/replicate). Following 8 h of feed and water withdrawal, the broilers were humanely euthanized by severing the jugular vein. The birds were scalded at 58 °C, de-feathered, eviscerated, and then chilled. The body parts, including the carcass, breast, thighs, abdominal fat, and internal organs (heart, liver, spleen, bursa of Fabricius, and pancreas), were dissected and weighed individually. All carcass parts and internal organs were expressed as percentages of the empty body weight using the following formula:

Cut yield (%) = (Weight of cut/Empty body weight) × 100.

### 2.5 Collection of blood samples for serum biochemical analyses

At the time of slaughter, approximately 10 mL of blood was collected from the vena jugularis and transferred into heparin tubes [Greiner Bio-One, Les Ulis, France, (23)]. The blood samples were then centrifuged (4,000 rpm at 10 °C for 10 min). Serum concentrations of immunoglobulin A (IgA; Otto Scientific, kit no: OttoBC146, Ankara, Turkey), and immunoglobulin M (IgM; Otto Scientific, kit no: OttoBC149, Ankara, Turkey) were determined using the MINDRAY-BS400 (Maharashtra, India) via the colorimetric method.

### 2.6 Ileal digestibility

The nutrient contents of the feeds used in the experiment were determined using the Weende analysis method (AOAC) (24) at the Chemical Analysis Laboratories of the Department of Animal Science, EÜZF The same methods were used for the nutrient analyses of ileal contents. During the trial period, 5 g/kg of TiO2 was added to the feed used in all groups, and on day 42, 10 chickens were slaughtered from each group to determine the dry matter, ash, nitrogen, and fat digestibility of the ileal contents. For this purpose, the TiO₂ content in the feed and manure was first determined using a spectrophotometric method; subsequently, the nutrient digestibility was calculated using the following regression equation based on the chemical analyses conducted on the feed and ileal contents (25):

Ileal Digestibility (%) = 100 − {(100 × TiO₂ in feed (%)/TiO₂ in ileal content (%)) × ((nutrient in ileal content (%)/nutrient in feed (%))}.

### 2.7 Microbiota in the cecum

Immediately following slaughter, the contents of the cecum were rapidly removed, flash-frozen in liquid nitrogen, and stored at −80 °C to determine the levels of *Lactobacillus* spp. and *Escherichia coli (E. coli)* using real-time PCR. Furthermore, DNA isolation was performed using the Qiagen QIAamp DNA Stool Mini Kit (Hilden, North Rhine-Westphalia, Germany). The concentrations and purity levels of the DNA extracted after isolation were determined using a Nanodrop device (Thermo Scientific, Wilmington, DE, USA). Primer designs for *Lactobacillus* spp. and *E. coli* microorganisms were prepared using the NCBI and ENSEMBLE gene banks (Table 2). The specifications of the prepared primers were examined using the BLAST program. Real-time PCR was performed using LightCycler® 480 SYBR Green I Master Mix (Roche Diagnostics, Mannheim, Germany). Amplification curves were generated using LightCycler® 480 II software to detect and quantify the target microorganisms. Cycle threshold (Ct) values were recorded, and the presence of positive samples was confirmed. The accuracy of the results was validated using Tm Calling Mode, and the data were evaluated accordingly. It was conducted at a private company in Istanbul.

**Table 2:** Primers used and sequences.

Microorganism	Primer name	Primer sequence 5′-3′
*E. coli*	Forward	GTCACAGTAACAAACCGTAACA
	Reverse	TCGTTGACTACTTCTTATCTGGA
*Lactobacillus* spp.	Forward	GAGGCAGCAGTAGGGAATCTTC
	Reverse	GGCCAGTTACTACCTCTATCCTTCTTC

*E. coli, Escherichia coli*.

### 2.8 Statistical analyses

Sample size estimation was conducted using GPower 3.1.9.7. The analysis indicated that a minimum of 60 animals per group (240 animals in total) was required to achieve a study power exceeding 80% at a 95% confidence interval, with a significance level of 0.05 and a medium effect size. For all data, the pen was considered to be the experimental unit. The normality of the data was assessed through the Kolmogorov–Smirnov test, while the homogeneity of variances was determined with Levene’s test. The data were analyzed using one-way ANOVA with IBM SPSS Statistics version 25. Significant differences among means were determined using Duncan’s multiple range test, with a significance threshold of *p* ≤ 0.05. Duncan’s multiple range test was chosen for *post hoc* comparisons due to its sensitivity in detecting differences among groups while controlling Type I errors, particularly with analyses involving a large number of groups (Table 3).

**Table 3:** Effect of humate and *Yucca* on growth performance in broiler chickens.

Performance parameters	Treatments				SEM	*p*
	C	H	Y	HY		
BW g						
Initial	38.04	38.27	38.55	38.68	0.074	0.102
21 days	861.96^d^	891.50^c^	918.78^b^	962.40^a^	8.553	< 0.001
42 days	2646.64^d^	2841.35^c^	2923.97^b^	2871.13^a^	29.043	< 0.001
BWG g						
Days 1–21	823.92^d^	853.23^c^	880.22^b^	923.72^a^	8.501	< 0.001
Days 21–42	1784.68^c^	1949.85^b^	2005.18^a^	2008.78^a^	21.83	< 0.043
Days 1–42	2608.50^d^	2803.09^c^	2885.41^b^	2932.45^a^	28.99	< 0.001
FI g						
Days 1–21	1301.04^d^	1320.48^c^	1340.24^b^	1350.36^a^	4.375	< 0.001
Days 21–42	3055.12^c^	3306.64^b^	3360.52^a^	3361.20^a^	30.05	< 0.001
Days 1–42	4356.16^d^	4627.12^c^	4700.76^b^	4711.56^a^	33.919	< 0.001
FCR						
Days 1–21	1.58^a^	1.55^b^	1.52^c^	1.47^d^	0.009	< 0.001
Days 21–42	1.71^a^	1.69^ab^	1.67^b^	1.67^b^	0.006	0.043
Days 1–42	1.67^a^	1.64^b^	1.63^bc^	1.61^c^	0.006	< 0.001

BW, body weight; BWG, body weight gain; FI, feed intake; FCR, feed conversion ratio. C, basal diet; H, basal diet + 300 g/kg humate; Y,100 g/kg Yucca; HY, basal diet + 300 g/kg humate + 100 g/kg Yucca a–d: Means in the same row with different superscripts indicates a significant difference (*p* < 0.05), while no superscript indicates no significant difference among groups (*p* > 0.05). SEM, standard error of the mean.

## 3 Discussion

In our study, the effects of humate and *Yucca* supplementation on live weight (BW) and live weight gain (BWG) in broiler chickens were clearly observed. These findings suggest that improvements in growth performance obtained from feed supplements containing humate and *Yucca* could enhance economic efficiency in the poultry industry. In the literature, the positive effects of humate and *Yucca* supplementation on growth performance are supported by various studies. For example, Hrncar et al. (26) reported that the combination of humate and probiotics improved live weight gain in broiler chickens by 5–7%. In this study, the mineral absorption-enhancing effect of humate and the regulatory role of probiotics in intestinal flora were proposed as the primary reasons for the increase in BWG. Similarly, Obianwuna et al. (27) reported that mixtures containing *Yucca* increased live weight gain in broiler chickens by up to 10%. The BW and BWG data from our study are largely consistent with these literature findings and confirm that the synergistic effects of humate and *Yucca* supplementation support growth performance.

The mineral absorption-enhancing effect of humate supports bone development and overall growth processes by improving the bioavailability of macrominerals such as calcium and phosphorus (19). The gut health-improving effect of *Yucca* supplementation contributes to BWG by enhancing nutrient absorption (28). However, the differences observed in BWG rates in our study compared to some studies in the literature may be due to variables such as the doses of humate and *Yucca* used, feed formulations, or environmental factors (e.g., coop temperature, stress factors). For example, Zhenglie et al. (29) emphasized that different dosages of *Yucca* combinations have variable effects on BWG and that determining the optimal dosage is critical. In this context, comparing the dosages in our study with those in the literature could serve as an important reference for future studies.

Feed intake (FI) and feed conversion ratio (FCR) are key parameters in broiler production, both economically and environmentally. In our study, significant improvements in feed intake and feed conversion ratio were observed in the humate and *Yucca*-supplemented groups. These findings suggest that humate and *Yucca* supplementation have the potential to reduce production costs by improving feed efficiency. There is strong evidence in the literature that *Yucca* supplementation improves feed conversion ratio. For example, Jun-Lin et al. (6) reported that the use of *Yucca schidigera* improved FCR by 4–6%. In this study, the supportive effect of *Yucca* on intestinal mucosa and its ammonia-reducing property were highlighted as factors that enhance feed absorption. Similarly, Samudovska and Demeterowa (19) noted that humate increases nutrient absorption by regulating the intestinal microbiota, thereby improving FCR.

The improvements in FCR observed in our study support the hypothesis that humate and *Yucca* supplementation improves nutrient absorption and metabolic efficiency by supporting intestinal health. In particular, it is known that *Yucca* supplementation increases intestinal villus height and reduces the colonization of pathogenic bacteria (28). This may be one of the underlying mechanisms behind the improvement in feed utilization efficiency. However, the effects on feed intake (FI) may be less pronounced in some cases compared to the control group. Bafundo et al., (9) noted that the indirect effects of *Yucca* supplementation on appetite may lead to small fluctuations in feed intake (9). The consistency of the FI data in our study with the literature indicates that humate and *Yucca* supplementation provide a balanced feed intake profile and that this supplementation increases economic efficiency. However, to better understand the variations in feed intake, it is recommended that the effects of other factors in feed composition (e.g., protein content or energy levels) be investigated in future studies.

The effect of humate and *Yucca* supplementation on overall performance in broiler chickens has yielded positive results in our study, both in terms of live weight gain and feed conversion ratio. These findings indicate that humate and *Yucca* supplementation not only have positive effects on individual parameters but also on overall production efficiency. In the literature, such supplements provide both economic and environmental advantages in poultry production. For example, Zhenglie et al. (29) reported that *Yucca* increases carcass yield and improves overall growth performance in broiler chickens. In this study, it was noted that humate’s mineral binding capacity and *Yucca*’s ammonia control capabilities enhance carcass quality and production efficiency. Similarly, Alfaro et al. (28) reported that *Yucca* supplementation contributed to environmental sustainability by reducing ammonia emissions and improving the poultry environment.

The data obtained in our study demonstrate that humate and *Yucca* supplementation not only improve performance parameters but also support environmental sustainability in the modern poultry industry. In particular, the reduction in ammonia emissions is a crucial factor that improves both animal welfare and environmental impacts. Additionally, Weiming et al. (30) noted that humate supplementation supports gut health by inhibiting the growth of pathogenic bacteria. This is a significant advantage for sustainable poultry farming and is consistent with the results of our study. The synergistic effects of humate and *Yucca* supplementation offer both economic and environmental benefits, making them a viable strategy for modern poultry production systems.

In our study, the effects of humate and *Yucca* supplementation on the intestinal microbiota of broiler chickens were evaluated, particularly in terms of *E. coli*, *lactic acid bacteria*, and *total bacterial* counts (Table 4). The results presented in the table indicate that humate (H), *Yucca* (Y), and the humate-*Yucca* combination (HY) significantly affected the intestinal microbiota (*p* < 0.001). These findings indicate that humate and *Yucca* supplementation have the potential to suppress the intestinal colonization of pathogenic bacteria. In the literature, it has been reported that *Yucca* supplementation inhibits the growth of pathogenic bacteria by reducing ammonia levels in the intestinal environment (28). Similarly, Kaya and Tuncer reported that humate reduces the proliferation of pathogens such as *E. coli* through its regulatory effect on intestinal pH. The decrease in *E. coli* counts in our study, particularly the lowest levels in the HY group, supports the notion that the synergistic effect between humate and *Yucca* enhances pathogen control efficacy.

**Table 4:** Effect of humate and *Yucca* on microbiota in broiler chickens (Item, log 10, CFU/g C).

Microorganism	Treatments				SEM	*p*
	C	H	Y	HY		
*E. coli*	5.38^a^	3.11^b^	2.72^c^	2.00^d^	0.244	< 0.001
*Lactic acid bacteria*	6.88^d^	7.43^c^	7.98^b^	8.14^a^	0.957	< 0.001
*Total bacteria*	7.80^d^	8.94^c^	9.23^b^	10.14^a^	0.162	< 0.001

C, basal diet; H, basal diet + 300 g/kg humate; Y, 100 g/kg Yucca; HY, basal diet + 300 g/kg humate + 100 g/kg Yucca a–d: Means in the same row with different superscript indicates a significant difference (*p* < 0.05), while no superscript indicates no significant difference among groups (*p* > 0.05). SEM, standard error of the mean.

*Lactic acid bacteria* are known to be beneficial microorganisms for intestinal health, and in our study, a significant increase in the number of these bacteria was observed in the humate and *Yucca*-supplemented groups. In particular, the HY group reached the highest level of *lactic acid bacteria*, indicating that the combination is more effective in positively regulating the intestinal microbiota. In the literature, it has been reported that *Yucca* supplementation supports the colonization of *lactic acid bacteria* by increasing intestinal villus height (31). Additionally, Mudronova et al. (32) reported that humate promotes the proliferation of beneficial bacteria. The increase in *lactic acid bacteria* in our study confirms the balancing effect of these additives on the intestinal flora and demonstrates their significant role in improving intestinal health.

An increase in *total bacterial* count was also observed in the humate and *Yucca*-supplemented groups. This increase may support nutrient absorption and metabolic efficiency by increasing the overall diversity and density of the gut microbiota. Zhenglie et al. (29) reported that *Yucca* increases the diversity of the gut microbiota and that this has a positive effect on growth performance. The increase in *total bacterial* count in our study, particularly at the highest level in the HY group, suggests that this combination has a synergistic effect in strengthening microbial balance. However, it is recommended that future studies confirm whether the increase in *total bacterial* count is due solely to beneficial bacteria or to the entire microbial population through more detailed molecular analyses (e.g., 16S rRNA sequencing).

These changes in the intestinal microbiota have indirect effects on the overall health and performance parameters of broiler chickens. A decrease in *E. coli* counts may contribute to improved intestinal health, reduced pathogen-induced stress, and consequently improved feed conversion ratio (FCR). Jun-Lin et al. (6) reported that *Yucca* supplementation reduces intestinal pathogens, increases feed absorption, and improves FCR by 4–6%. The decrease in *E. coli* counts in our study is consistent with these findings and suggests that the humate-*Yucca* combination may improve economic efficiency by promoting intestinal health.

The increase in *lactic acid bacteria* may improve nutrient absorption and immunological response by strengthening intestinal barrier function. Bafundo et al. (9) reported that the combination of *Yucca* and quillaja supports intestinal health by increasing IgA and IgM levels (9). The increase in *lactic acid bacteria* in our study may support the immune system through a similar mechanism, thereby increasing the disease resistance of broiler chickens. Additionally, the increase in *total bacterial* count may support metabolic processes by enhancing the overall diversity of the intestinal microbiota. Obianwuna et al. (27) noted that mixtures containing *Yucca* improved metabolic parameters (e.g., T3 and T4 levels) and that this effect was associated with the gut microbiota. The microbiota changes observed in our study may be associated with such metabolic improvements, and their contributions to growth performance require further detailed investigation.

The positive effects of humate and *Yucca* supplementation on the gut microbiota offer significant advantages for sustainable production in the poultry industry. The reduction of pathogenic bacteria such as *E. coli* has the potential to reduce antibiotic use, thereby supporting both animal welfare and environmental sustainability. Mudronova et al. (32) emphasized that humate supplementation reduces the need for antibiotics by inhibiting pathogenic bacteria. Additionally, the ammonia-reducing effect of *Yucca* supplementation improves the poultry environment, contributing to environmental sustainability (28). The microbiome changes observed in our study indicate that the combination of humate and *Yucca* provides both economic and environmental benefits. In particular, the HY group yielding the best results in all parameters (reduction in *E. coli*, increase in lactic acid bacteria, and *total bacteria*) suggests that this combination may be favored in practical applications. This mechanism is explained by Mudronova et al. (32) as follows: By balancing gut flora, it significantly reduces pathogenic bacteria (e.g., Enterobacteriaceae), lowering microbial pressure and promoting a slight increase in beneficial bacteria (e.g., lactic acid bacteria). This enhances nutrient absorption and strengthens the gut barrier by humate. In the mechanism described by Alghirani et al. (31), the working principle of *Yucca* is explained as follows: It reduces microbial pressure by decreasing pathogenic bacteria (e.g., *Enterococcus faecalis*) and promotes the growth of beneficial bacteria (e.g., Lactococcus, Streptococcus, Parabacteroides). This alters beta diversity, enriching the gut microbial composition, and reduces the risk of diarrhea by binding toxins such as ammonia.

This study evaluated the effects of humate and *Yucca schidigera* supplementation on ileal digestibility in broiler chickens at 42 days of age. The table presents the effects of humate (H), *Yucca schidigera* (Y), and their combination (HY) on the digestibility of crude fat, crude protein, and organic matter. The results indicate that both humate and *Yucca schidigera* supplementation enhanced ileal digestibility, with the combination (HY) yielding the most significant effect (*p* < 0.001). These findings support the potential of these substances to improve digestive processes and their application as alternative feed additives in broiler production. According to the table, crude fat digestibility was highest in the HY group (88.08%) and lowest in the control group (85.81%) (*p* < 0.001). The *Yucca schidigera* group (87.26%) exhibited higher digestibility compared to the humate group (86.46%), reflecting *Yucca schidigera*’s ability to enhance lipid absorption in the intestinal environment. *Yucca schidigera*, due to its saponin content, may increase intestinal permeability and facilitate fat absorption (33). This effect may be attributed to an increased surface area of intestinal villi or modulation of microbial activity (34). Humate, on the other hand, supports digestion by optimizing intestinal pH and regulating microbial balance (26). However, the effect of humate alone appears to be more limited compared to *Yucca schidigera*, highlighting the latter’s specific influence on lipid metabolism. The combination group (HY) demonstrated a synergistic effect, achieving the highest crude fat digestibility. This is consistent with findings by (23), which reported improved digestibility with a combination of humate and enzymes. Regarding crude protein digestibility, the HY group recorded the highest value (87.11%), while no significant difference was observed between the control (85.13%) and humate (84.81%) groups (*p* < 0.001). The *Yucca schidigera* group (86.55%) outperformed both the control and humate groups.

In the literature, the supportive effects of humate and *Yucca* supplementation on digestive processes have been reported in various studies. For example, Mudronova et al. (32) reported that humic substances enhance nutrient absorption and improve organic matter digestibility by strengthening the intestinal mucosa. Similarly, Mohammed et al. (34) demonstrated that *Yucca schidigera* supplementation improves intestinal environment and increases fat and protein digestibility. The increases in crude fat, crude protein, and organic matter digestibility observed in our study are consistent with these findings and support the hypothesis that humate and *Yucca* enhance digestive enzyme activity by supporting intestinal health. The highest digestibility values in the HY group indicate that the combined use of humate and *Yucca* creates a stronger effect than their individual use. This synergistic effect may result from the combination of humate’s mineral absorption-enhancing property and *Yucca*’s intestinal villus structure-improving effect (27, 35).

Table 5 shows that crude fat digestibility improved significantly in the humate and *Yucca*-supplemented groups (*p* < 0.001). The HY group achieved the highest crude fat digestibility value, indicating that this combination is particularly effective in enhancing lipid absorption. In the literature, it has been reported that *Yucca* supplementation supports fat absorption by increasing intestinal villus height (31). Additionally, Alfaro et al. (28) reported that *Yucca schidigera* improves digestive processes and increases lipid absorption by reducing ammonia levels in the intestinal environment. The increase in crude fat digestibility in our study may be related to these mechanisms. The effect of humate in enhancing mineral bioavailability may be an indirect factor supporting fat digestibility, as the absorption of minerals such as calcium and magnesium can increase the activity of enzymes involved in lipid metabolism (19). The significant improvement in the HY group indicates that humate and *Yucca* play a synergistic role in optimizing the intestinal environment. However, a more detailed examination of the effects of different combinations of humate and *Yucca* dosages (e.g., other than 300 g/kg humate and 100 g/kg *Yucca*) on crude fat digestibility is recommended for future studies.

**Table 5:** Effect of humate and *Yucca* on ileal digestibility (42 day) in broiler chickens.

Nutrient of digestibility	Treatments				SEM	*p*
	C	H	Y	HY		
Crude fat (%)	85.81^d^	86.46^c^	87.26^b^	88.08^a^	0.131	< 0.001
Crude protein (%)	85.13^c^	84.81^c^	86.55^b^	87.11^a^	0.146	< 0.001
Organic matter (%)	83.43^d^	83.75^c^	84.20^b^	85.51^a^	0.104	< 0.001

C, basal diet; H, basal diet + 300 g/kg humate; Y, 100 g/kg Yucca; HY, basal diet + 300 g/kg humate + 100 g/kg Yucca a–d: Means in the same row with different superscript indicates a significant difference (*p* < 0.05), while no superscript indicates no significant difference among groups (*p* > 0.05). SEM, standard error of the mean.

Crude protein digestibility and organic matter digestibility were significantly increased in the humate and *Yucca*-supplemented groups (*p* < 0.001). The HY group exhibited the highest values for both parameters, indicating that the combined use provides a superior effect on protein and organic matter absorption. In the literature, humate has been reported to support protein digestion by strengthening the intestinal mucosa (23). This study reported that humic substances regulate intestinal pH, increase the activity of proteolytic enzymes, and improve protein absorption. Additionally, Zhenglie et al. (29) reported that *Yucca* improves intestinal health and increases the digestibility of organic matter.

In our study, the effects of humate (H: 300 mg/kg), *Yucca* (Y: 100 mg/kg), and the humate-*Yucca* combination (HY) on blood parameters (IgA, IgM, T3, T4) in broiler chickens were evaluated. The results presented in the table show that the humate and *Yucca*-supplemented groups (H, Y, HY) exhibited significant improvements in IgA, IgM, T3, and T4 levels compared to the control group (C) (*p* < 0.001 and *p* < 0.002). In particular, IgA (C: 9.47 mg/dL; HY: 10.98 mg/dL), IgM (C: 7.15 mg/dL; HY: 8.28 mg/dL), T3 (C: 1.21; HY: 1.41), and T4 (C: 9.05; HY: 10.09) levels reached the highest values in the HY group. These findings indicate that humate and *Yucca* supplementation has supportive effects on immune response and metabolic processes. In the literature, the positive effects of humate and *Yucca* supplementation on immune and metabolic parameters are supported by various studies. For example, Zhenglie et al. (29) reported that *Yucca* increases IgA and IgM levels. Similarly, Obianwuna et al. (27) reported that mixtures containing *Yucca* increased IgA and IgM levels by 12% and T3 and T4 levels by 10%. The results of our study are consistent with these findings and confirm the potential of humate and *Yucca* to improve immune and metabolic processes.

Table 6 shows that in the humate and *Yucca*-supplemented groups, (IgA and IgM) levels in the humate and *Yucca*-supplemented groups. The HY group exhibited the highest levels of IgA and IgM, but the differences between the HY group and the H and Y groups were not statistically significant (*p* > 0.05). This indicates that humate and *Yucca* supplementation are effective in enhancing the immune response both alone and in combination. The literature indicates that *Yucca* supplementation improves mucosal immunity (IgA) by supporting the intestinal mucosa. The increases in IgA and IgM levels in our study are consistent with these findings and suggest that humate and *Yucca* enhance the immune response by supporting intestinal health. While the slightly elevated values in the HY group suggest the presence of a synergistic effect, the lack of statistical significance of this difference suggests that the dosage or duration of application may need to be optimized. In the study by Mudronová, et al. (32) the effect on immunity is described as follows: It stimulates phagocytic activity and the engulfment capacity of phagocytes, enhancing cellular immunity by increasing the CD4+: CD8 + lymphocyte ratio. Additionally, it upregulates the expression of the MUC-2 (mucin 2) gene, which protects the intestinal mucosa, but has a neutral effect on IgA and certain antimicrobial peptides (e.g., AvBD2). Through its anti-inflammatory and antioxidant properties, it can reduce pro-inflammatory cytokines such as TNF-*α*. In the study by Alghirani et al. (31), the effect of *Yucca* on immunity is described as follows: It enhances humoral immunity by increasing serum IgM and total protein levels. Through polyphenols, it exerts anti-inflammatory effects, improves antioxidant status, and reduces liver enzymes (e.g., ALT). By improving gut morphology (increasing villus height and reducing crypt depth), it indirectly strengthens the immune barrier.

**Table 6:** Effect of humate and *Yucca* on some blood parameters in broiler chickens.

	Treatments				SEM	*p*
	C	H	Y	HY		
IgA mg/dL	9.47 ^b^	10.50 ^a^	10.78^a^	10.98^a^	0.06	< 0.001
IgM mg/dL	7.15 ^b^	8.20^a^	8.26^a^	8.28^a^	0.04	< 0.001
T3	1.21^b^	1.38^a^	1.40^a^	1.41^a^	0.01	< 0.001
T4	9.05 ^b^	10.02 ^a^	10.08 ^a^	10.09 ^a^	0.05	0.002

IgA Immunoglobulin A, IgM Immunoglobulin M. T3 Triiodothyronine, T4 Thyroxine, C, basal diet; H, basal diet + 300 g/kg humate; Y, 100 g/kg Yucca; HY, basal diet + 300 g/kg humate + 100 g/kg Yucca a–b: Means in the same row with different superscript indicates a significant difference (*p* < 0.05), while no superscript indicates no significant difference among groups (*p* > 0.05). SEM, standard error of the mean.

Levels of T3 (*p* < 0.001) and T4 (*p* < 0.002) were significantly increased in the humate and *Yucca*-supplemented groups compared to the control group. The HY group showed the highest values for T3 and T4 levels, but the differences between it and the H and Y groups were not statistically significant (*p* > 0.05). These findings suggest that humate and *Yucca* supplementation supports metabolic processes and increases thyroid hormone production. Additionally, Obianwuna et al. (27) reported that *Yucca*-containing mixtures increased T3 and T4 levels by 10% and that this effect was associated with the relationship between gut health and metabolic efficiency. The increases in T3 and T4 levels in our study are consistent with these findings and strengthen the hypothesis that humate and *Yucca* improve gut health, thereby enhancing nutrient absorption and supporting metabolic processes. The slightly elevated values in the HY group suggest the presence of a synergistic effect, but the lack of statistical significance suggests that this effect may become more pronounced with extended duration or varying dosages.

## 4 Results

Humate and *Yucca* supplementation significantly enhanced growth performance in broiler chickens across all measured periods. At 21 and 42 days, body weight (BW) was highest in the humate-*Yucca* combination (HY) group, followed by *Yucca* (Y), humate (H), and control (C) groups, with significant differences (*p* < 0.001). Body weight gain (BWG) followed a similar trend, with HY and Y groups outperforming H and C from days 1–21 and 1–42 (*p* < 0.001), while HY and Y were comparable from days 21–42 (*p* < 0.043). Feed intake (FI) increased progressively from C to HY across all periods (p < 0.001), with HY and Y showing similar intake from days 21–42. Feed conversion ratio (FCR) improved significantly with supplementation, with HY achieving the lowest (most efficient) FCR across all periods (p < 0.001), followed by Y, H, and C. No significant differences were observed in initial BW (*p* = 0.102). These results indicate that humate and *Yucca*, particularly in combination, promote superior growth, feed efficiency, and feed intake in broilers.

Humate and *Yucca* supplementation significantly altered the intestinal microbiota in broiler chickens. The humate-*Yucca* combination (HY) most effectively reduced *E. coli* counts, followed by *Yucca* (Y) and humate (H), with all supplemented groups showing lower counts than the control (C) (*p* < 0.001). Conversely, *lactic acid bacteria* counts were highest in the HY group, followed by Y, H, and C, with significant differences across all groups (*p* < 0.001). *Total bacterial* counts also increased progressively from C to HY, with HY exhibiting the highest counts (*p* < 0.001). These findings suggest that humate and *Yucca*, particularly in combination, promote a favorable shift in gut microbiota by reducing harmful *E. coli* while enhancing beneficial *lactic acid bacteria* and overall bacterial populations.

Humate and *Yucca* supplementation significantly improved ileal digestibility in broiler chickens at 42 days. The humate-*Yucca* combination (HY) yielded the highest digestibility for crude fat, crude protein, and organic matter, followed by *Yucca* (Y), humate (H), and the control (C) (*p* < 0.001). For crude fat and organic matter, all treatment groups differed significantly, with HY showing the greatest improvement. For crude protein, HY and Y outperformed H and C, with no significant difference between H and C (*p* < 0.001). These results indicate that humate and *Yucca*, particularly in combination, enhance nutrient digestibility in the ileum, with HY consistently showing the most pronounced effects.

Humate and *Yucca* supplementation significantly influenced blood parameters in broiler chickens. The humate-*Yucca* combination (HY), *Yucca* (Y), and humate (H) groups showed significantly higher levels of IgA, IgM, T3, and T4 compared to the control (C) group (*p* < 0.001 for IgA, IgM, T3; *p* < 0.002 for T4). No significant differences were observed among HY, Y, and H groups for any parameter, indicating comparable efficacy of the supplements. These findings suggest that humate and *Yucca*, individually or in combination, enhance immune and thyroid hormone levels in broilers, with no additive effect observed in the HY group compared to H or Y alone.

## 5 Conclusion

Our study demonstrated that humate (300 mg/kg), *Yucca* (100 mg/kg), and the humate-*Yucca* combination (HY) significantly improved growth performance, intestinal microbiota, ileal digestibility, and blood parameters in broiler chickens. The increases observed in live weight gain and feed conversion ratio indicate that the addition of humate and *Yucca* supports growth performance. In the intestinal microbiota, a significant reduction in *E. coli* counts, along with an increase in lactic acid bacteria and *total bacterial* counts in the intestinal microbiota, indicates that these additives improve gut health and control pathogens. In ileal digestibility, the highest values observed in the HY group for crude fat crude protein, and organic matter confirm increased nutrient absorption. Increases in blood parameters such as IgA, IgM, T3, and T4 levels indicate improved immune and metabolic processes. These results demonstrate that the combination of humate and *Yucca* is an effective strategy for enhancing performance, health, and productivity in broiler chicken production. Following the ban on antibiotics, the increasing trend toward natural feed additives has led to the use of various feed additives. Our study has demonstrated that humate and *Yucca* yield successful results, while their combination shows even greater potential for enhanced outcomes. Future studies should investigate their effects in greater depth.

## Data Availability

The original contributions presented in the study are included in the article/supplementary material, further inquiries can be directed to the corresponding author.
